# A probiotic approach identifies a Treg-centred immunoregulation *via* modulation of gut microbiota metabolites in people with multiple sclerosis and healthy individuals

**DOI:** 10.1016/j.ebiom.2025.105743

**Published:** 2025-05-12

**Authors:** Constantin Träger, Maria Kaiser, David Freudenstein, Simon Heckscher, Katja Dettmer, Peter J. Oefner, Gerhard Liebisch, Andreas Hiergeist, André Gessner, De-Hyung Lee, Klemens Angstwurm, Ralf A. Linker, Stefanie Haase

**Affiliations:** a Department of Neurology, University Hospital Regensburg, Regensburg, Germany; b Institute of Functional Genomics, University of Regensburg, Regensburg, Germany; c Institute of Clinical Chemistry and Laboratory Medicine, University Hospital Regensburg, Regensburg, Germany; d Institute of Clinical Microbiology and Hygiene, University Medical Center Regensburg, Regensburg, Germany

**Keywords:** Probiotic supplementation, Multiple sclerosis, Immunomodulation, Enhanced Treg suppressive capacity, Indole-3-actetic acid, AHR signalling

## Abstract

**Background:**

Modulation of the gut microbiota composition has been suggested as a potential disease modifying therapy in immune-mediated diseases such as multiple sclerosis (MS). However, a conclusive mechanism linking gut microbiota modulation to peripheral immune responses has remained elusive so far.

**Methods:**

In this exploratory cohort study, people with MS (pwMS) and healthy controls (HC) supplemented a lactobacilli-rich probiotic for two or six weeks and were additionally investigated six weeks after the last intake. Immune cell phenotyping was performed in blood samples, complemented by mRNA expression analysis, serum cytokine measurements, and Treg suppression assays. Besides gut microbiota composition analysis, metabolite production was investigated in stool and serum. Links between metabolites and peripheral immune system were investigated in *in vitro* T cell differentiation assays.

**Findings:**

In peripheral blood, Treg cells increased in both groups, while Th1 cells were significantly reduced in pwMS. This promotion of a regulatory immunophenotype was complemented by increased concentrations of IL-10 in serum and higher expression of *IL10* and *CTLA4*. Functional assays revealed an enhanced suppressive capacity of Treg cells due to the probiotic intervention. The tryptophan metabolite indole-3-acetate (IAA) increased in stool and serum samples of pwMS during the probiotic intake. *In vitro*, IAA specifically enhanced the formation of IL-10 secreting T cells together with *CYP1a1* expression. This effect was blocked by addition of an aryl hydrocarbon receptor (AHR) inhibitor.

**Interpretation:**

A lactobacilli-enriched probiotic promotes a regulatory immunophenotype in pwMS, probably by enhancing AHR agonists in the gut. It may be of interest as add-on therapy in immune-mediated diseases such as MS.

**Funding:**

This study has in part been funded by 10.13039/100008792Novartis Pharma GmbH and 10.13039/501100002347BMBF grant no. 01EJ2202B.

**Research in context**

**Evidence before this study**

Functional analysis revealed that commensal gut bacteria can influence peripheral immune cells either directly or *via* production of immunomodulatory metabolites. Moreover, mounting evidence has shown an altered gut microbiota composition in pwMS, potentially fostering a pro-inflammatory immune phenotype. It thus seems promising that modulation of the gut microbiota represents an interesting option for a beneficial immunomodulation in MS. Here, the supplementation of probiotics rich in lactobacilli might be of potential interest. Lactobacilli have previously been credited with the ability to induce IL-10 producing Treg cells in EAE diseased mice and an amelioration of EAE symptoms. Favourable effects of probiotic supplementation have also been observed in pwMS. However, it remains unclear how probiotic intervention affects the broader immune system *via* gut metabolites in patients suffering from MS, and whether it might serve as suitable candidate for add-on therapy.

**Added value of this study**

This study offers a comprehensive analysis of peripheral blood immune cells and cytokines as well as regulatory T cell function, complemented by tryptophan-related metabolite analysis and functional assays in pwMS and HC before and after a probiotic intervention. We here show that probiotic intake enhances functionally active Treg cells even in immune-depleted pwMS. Importantly, and unlike previous studies investigating probiotic supplementation in MS, we additionally performed mechanistic studies in the blood and the gut and identified a potential mode of action. Our data clearly connect probiotic supplementation with enhanced tryptophan metabolite production in the gut and the induction of an AHR-mediated regulatory pathway in T cells.

**Implications of all the available evidence**

Our data identified possible future targets for the therapy of neuroinflammatory disease such as MS. Since the gut microbiota or its metabolites are currently not the primary therapeutic target for MS, we expect that probiotic modulation in MS may be a complementary approach to the existing baseline disease-modifying therapies by strengthened Treg-mediated immunoregulation.

## Introduction

The cause of MS is most likely the result of genetic susceptibility, epigenetic and post-genomic regulation, and–most critically–environmental factors,^1^ including EBV infection,^2^^,^^3^ low Vitamin-D levels,^4^ smoking,^5^ and possibly nutrition.^6^ The pathology of the disease includes an inflammatory process resulting in the destruction of myelin sheets, impaired neural transmission, and the formation of multiple lesions in the CNS, which eventually turn into glial scars.^7^ At a microscopic level, pro-inflammatory cells foster autoimmune destruction *via* cytokines and direct cellular interaction. B cells, Th1 cells, and Th17 cells are considered pro-inflammatory, while Treg and regulatory B cells (Breg) may alleviate the disease.^8^^,^^9^ Treg deficiency worsened the clinical course in experimental autoimmune encephalomyelitis (EAE), an animal model of MS, while strong functionality suppressed autoimmune reactions,^10^^,^^11^ potentially involving IL-10 signalling.^12^ A reduced suppressive capacity of Treg cells has also been observed in people with MS (pwMS).^13^ Currently available pharmaceutics typically suppress pro-inflammatory cells, while there is no existing therapy that directly supports immunoregulation.

The geographic distribution of MS may be connected to environmental influences, especially Vitamin-D and nutrition. Adding to this observation, the gut microbiota is considered as a new (environmental) risk factor for MS.^14^^,^^15^ The gut microbiota may contribute to MS pathology *via* direct interactions with immune cells or the production of immunomodulatory metabolites,^16^ also referred to as microbiota-gut-brain axis. Appropriately, an altered microbiome composition as observed in pwMS^17^, ^18^, ^19^, ^20^ was recently linked to a selective increase of Th17 cells in the small intestine.^21^ In contrast, bacterial strains that were shown to enhance the differentiation of regulatory immune cells are decreased or even not-existent in the microbiota of pwMS,^22^ indicating a pro-inflammatory environment in the gut of pwMS. It thus seems promising that modulation of the gut microbiota represents an interesting therapeutic option for immunomodulation in MS. Short-chain fatty acids (SCFAs), which physiologically derive from microbiota fermenting carbohydrates in the gut,^23^ may boost Treg and inhibit Th1 and Th17 mediated inflammation.^24^^,^^25^ Moreover, oral supplementation of the SCFA propionic acid beneficially influenced the gut microbiota and exerted positive effects on the clinical course of MS.^25^ Our own research on myelin oligodendrocyte glycoprotein (MOG)-induced EAE, an animal model mimicking aspects of MS, further supported the potential of microbiota modulation *via* probiotic lactobacilli supplementation.^26^ Coinciding with other studies,^27^^,^^28^ targeted probiotic treatment with lactobacilli alleviated EAE. We identified a potential involvement of the faecal tryptophan metabolite indole-3-lactic acid (ILA) produced by lactobacilli,^29^ which is implicated in the suppression of CNS autoimmunity *via* aryl hydrocarbon receptor (AHR) activation.^30^ Along this line, probiotic lactobacilli supplementation could correct a lactobacilli dysbalance in the human gut after high salt intake, thus counteracting a high-salt associated increase of Th17 cells.^26^ These data indicated that re-shaping of the gut microbiota by probiotic supplementation might constitute a promising tool to modulate the immune response in diseases involving a Th17 immunopathology. Indeed, first trials revealed a beneficial effect on clinical and inflammatory markers in pwMS after a probiotic intervention.^31^, ^32^, ^33^, ^34^, ^35^ However, immunological findings have been insufficiently addressed and a conclusive mechanism linking gut microbiota modulation to peripheral immune responses has remained elusive so far. Our study supports the beneficial effect of probiotic supplementation in pwMS, and, for the first time, clearly connects microbial metabolites to specific changes in MS patients' immune system involving AHR-signalling.

## Methods

### Ethics

This study was approved by the appropriate institutional review boards at the University of Regensburg and the Friedrich-Alexander University Erlangen-Nürnberg according to the principles of the Declaration of Helsinki (approval numbers: 21-2307-101 and 352_17B). All participants gave their written informed consent.

### Study participants

All study participants were recruited between August 2020 and October 2021. Our study examined male and female participants, and similar findings are reported for both sexes. We invited all pwMS who were being treated for active MS according to the 2013 revisions on the clinical course of MS that were initially diagnosed according to the 2017 McDonald criteria, EDSS-scored, and treated by attending physicians at the Department of Neurology. Healthy volunteers (HC; n = 41) were recruited at the University of Regensburg. We included all pwMS and HC that did not match exclusion criteria (hypertension, chronic kidney failure, on-going oncologic disease, severe gastrointestinal conditions, acute or chronic infection, current post-surgical rehabilitation, alcohol or drug abuse, vaccination within the last 6 weeks, relapse within the last 6 months, regular probiotic intake or the intake of nutritional supplements besides Vitamin D), were at least 18 years old, were able to understand and follow the study protocol, and were both willing and able to travel to our clinic. PwMS were recruited without special focus on their individual medication. Most patients (82%) were treated intravenously with anti-CD20 antibodies (Table 1). Here, only patients who had received their third cycle of B-cell depleting therapy (one year of treatment) without relapse in the six months prior to the probiotic intervention were included in the study. A-priori sample size calculations for potential effects between time points within HC and pwMS (using G.Power 3.1; matched-pairs Wilcoxon signed-rank-test, α-error 0.05, intended minimum test power 0.8) identified a minimum sample size of n = 12 participants per group to identify potential large effects (d = 0.8) and an optimal sample size of n = 28 participants per group to identify potential medium effects (d = 0.5). Post-hoc analysis for potential large effects (d = 0.8, α 0.05) revealed an achieved test power of 0.99 in HC (n = 41) and 0.99 in pwMS (n = 28).

**Table 1:** Clinical characteristics of HC and pwMS.

	2 weeks		6 weeks + washout	
	HC	pwMS	HC	pwMS
Female/male [n]	26/15	13/15	10/7	4/11
Median age [y (range)]	23 (21–63)	45 (21–64)	27 (21–63)	48 (31–64)
Median BMI ± SD [kg/m^2^]	22 ± 4	27 ± 4	23 ± 3	26 ± 3
RRMS [% of MS]	–	78	–	75
EDSS [median points (range)]	–	3 (1–7)	–	3 (0–6)
Disease duration [median y (range)]	–	12 (1–33)	–	10 (1–27)
B cell depleted [% of MS]	–	82	–	70

### Probiotic intervention

Study participants were instructed to supplement two sachets of Vivomixx™ three times per day (equal to a total of 2.7 trillion CFU daily) dissolved in a cool, non-carbonated, non-alcoholic drink. All study participants supplemented Vivomixx™ for at least two weeks, a group of 15 pwMS and 17 HC continued the probiotic intake for additional six weeks. This subgroup consisted of voluntary patients and controls who chose to continue participating in the study due to easy logistics (such as short travel distance to the clinic, flexible working hours) and self-motivation. PwMS supplemented the probiotic add-on to their existing therapy. Vivomixx™ was provided by Mendes S. A. (Luganos, Switzerland). The probiotic contains *Lactobacillus acidophilus* DSM24735®/NCIMB30442, *Lactobacillus plantarum* DSM24730®/NCIMB30437, *Lactobacillus paracasei* DSM24733®/NCIMB30439, *Lactobacillus delbrueckii* subsp. *bulgaricus* DSM24734®/NCIMB 30440, *Streptococcus thermophilus* DSM24731®/NCIMB30438, *Bifidobacterium breve* DSM24732®/NCIMB30441, *Bifidobacterium longum* DSM24736®/NCIMB30435, and *Bifidobacterium infantis* DSM24737®/NCIMB30436. Participants were asked to return unused sachets for compliance control.

A detailed list of all reagents used in this study are listed in Suppl. Table S3.

### Blood sample acquisition and handling

We conducted appointments at the Department of Neurology of the University Hospital Regensburg prior to the probiotic intake, after two, and after six weeks as well as six weeks after the termination of probiotic supplementation. At each time point, the last probiotic intake occurred 12 h before the stool and blood collection. Sample collection occurred during the outpatient examination in the morning hours. Peripheral venous blood samples were drawn and transported at room temperature (RT) for further processing. Routine clinical markers were analysed by the central laboratory at the medbo Bezirksklinikum Regensburg. Serum was extracted after centrifugation from whole blood in a Serum Gel CAT Monovette (Sarstedt) and frozen immediately at −80 °C. Whole blood samples were stored in S-Monovettes EDTA KE (Sarstedt) on a rolling mixer at RT until the isolation of peripheral blood mononuclear cells within 24 h.

### Immune cell phenotyping in whole blood

Whole blood was incubated at RT in the dark with 0.1 M NH_4_Cl (Sigma–Aldrich) to lyse erythrocytes. Erythrocytic fragments were removed by washing with ice cold FACS-buffer (containing MACS BSA Stock Solution (Miltenyi Biotec), Auto MACS Rinsing Solution with EDTA and PBS (Miltenyi Biotec)). Non-specific Fc mediated interactions were blocked by the addition of Fc-receptor blocking agent (MiltenyiBiotec; RRID:AB_2892112). Cells were stained for 45 min at 4 °C using fluorochrome-conjugated antibodies or respective isotype controls as listed in Table 2.

**Table 2:** Antibodies and isotype controls used for flow cytometry analysis.

Antigen	Host, isotype	Conjugate	Clone	Vendor	RRID
CD3	Mouse, IgG1, κ	eFluor 450	UCHT1	eBioscience	AB_1518798
CD4	Mouse, IgG1, κ	PerCP	SK3	Biolegend	AB_2563326
CD4	Mouse, IgG1, κ	Pacific blue	SK3	Biolegend	AB_2228841
CD8	Mouse, IgG1, κ	APC	SK1	BD Bioscience	AB_2868803
CD14	Mouse, IgG2b, κ	APC	MϕP9	BD Bioscience	AB_2868813
CD16	Mouse, IgG1, κ	FITC	eBioCB16	eBioscience	AB_823123
CD19	Mouse, IgG1, κ	PE-Cy7	HIB19	Biolegend	AB_314245
CD25	Mouse, IgG1, κ	PE	2A3	BD Bioscience	AB_2783790
CD45RO	Mouse, IgG2a, κ	PE-Cy7	UCHL1	eBioscience	AB_10718534
CD56	Mouse IgG1, κ	PE	HCD56	Biolegend	AB_604093
CCR7	Rat, IgG2a, κ	PE	3D12	eBiosciences	AB_10670625
Foxp3	Rat, IgG2a, κ	APC	PCH101	eBioscience	AB_1603280
Foxp3	Mouse, IgG1, κ	PE	206D	Biolegend	AB_492986
IFN-γ	Mouse, IgG1, κ	APC	B27	Biolegend	AB_315443
IL-4	Rat, IgG1, κ	FITC	MP4-25D2	Biolegend	AB_315126
IL-17A	Mouse, IgG1, κ	PE	eBio64DEC17	eBioscience	AB_1724138
**Isotype controls**					
Mouse IgG1, κ		APC	MOPC-21	BD Bioscience	AB_398613
Mouse IgG1, κ		eFluor 450	P3.6.2.8.1	eBioscience	AB_1271992
Mouse IgG1, κ		FITC	P3.6.2.8.1	eBioscience	AB_10596964
Mouse IgG1, κ		Pacific Blue	MOPC-21	Biolegend	AB_2923473
Mouse IgG1, κ		PE	MOPC-21	BD Bioscience	AB_396091
Mouse IgG1, κ		PE-Cy7	MOPC-21	Biolegend	AB_2861433
Mouse IgG2b, κ		APC		BD Bioscience	AB_2869669
Rat IgG1, κ		FITC	RTK2071	Biolegend	AB_326511
Rat IgG2a, κ		APC	eBR2a	eBioscience	AB_470181
Rat IgG2a, κ		PE	eBR2a	eBioscience	AB_1834380

Stained cells were analysed on a BD FACS Canto II flow cytometer. FCS files were analysed with FlowJo v10.6.2.

### Isolation of peripheral blood mononuclear cells

Peripheral blood mononuclear cells (PBMCs) were isolated *via* density gradient centrifugation using Pancoll human (PAN-Biotech). Live cells were counted after Acridine Orange Propidium Bromide staining (Logos Biosystems) and adjusted to a cell concentration of 1 × 10^6^ cells per ml in R10 medium containing RPMI Medium 1640 with GlutaMAX™ (Gibco *via* Life Technologies), 200 mM l-Glutamine (Pantm Biotech), Penicillin-Streptomycin P4458 (Sigma–Aldrich), 100 mM Sodium pyruvate solution (Sigma–Aldrich), non-essential amino-acids (Gibco *via* Life Technologies), and 10% foetal bovine serum (Gibco *via* Life Technologies). PBMCs were directly used for intracellular cytokine staining or processed for RNA isolation and cryopreservation for later experiments. In brief, 1 × 10^6^ PBMCs were lysed in 350 μL RNeasy Plus lysis buffer (Qiagen) and stored at −80 °C until RNA isolation. For cryopreservation, 1 × 10^6^ PBMCs were washed, re-suspended in 900 μL foetal bovine serum (Gibco *via* Life Technologies) and mixed with 100 μL dimethyl sulfoxide (ATCC). Cells were frozen slowly to −80 °C and stored at −196 °C for later experiments.

### Intracellular cytokine staining and analysis

1 × 10^6^ freshly isolated PBMCs were stimulated with ionomycin (0.75 mg/mL in DMSO, Sigma–Aldrich) and PMA (β = 1 mg/mL in R10, Sigma–Aldrich) together with Golgi-Stop Solution (containing Monensin, BD Biosciences) for 3 h at 37 °C. Dead cells were excluded by Fixable Viability Dye eFluor® 780 (eBioscience). Non-specific Fc mediated interactions were blocked by the addition of Fc-receptor blocking agent (MiltenyiBiotec; RRID: AB_2892112). For surface staining, cells were stained with CD4 pacific blue (#SK3, BioLegend; Table 2) for 30 min in FACS buffer. Cells were then fixed and made permeable using the FOXP3 Fix/Perm Buffer Set (eBioscience) according to the manufacturer's instructions. Intracellular cytokines were stained with the respective fluorochrome conjugated antibodies or isotype controls for 45 min at 4 °C as listed in Table 2. Stained cells were analysed in a BD FACS Canto II flow cytometer. FCS files were analysed with FlowJo v10.6.2.

### Real-time polymerase chain reaction (qPCR)

Gene expression was analysed by real-time PCR. Total RNA was isolated using the RNase Mini Kit #A2791 (Qiagen) following the manufacturer's instructions. RNA yield was quantified by absorbance measurements at 260 nm. Reverse transcription was performed by using the GoScript Reverse Transcription Mix Oligo(dT) (Promega). PCR reactions were performed at a 5 μl scale on a qTower 2.0 real-time PCR System (Analytic Jena, Jena, Germany) in triplicates. Relative quantification was performed by the ΔΔCT method, normalising target gene expression on GPADH as housekeeping gene.^36^

### Cytokine measurements

Cytokine concentrations in serum samples were analysed by enzyme linked immunosorbent assay (ELISA) for IL-10, IFNγ, IL-17A, IL-23, and IL-6 (all BioLegend) according to the manufacturers' instructions.

### Suppression assay

PBMCs were isolated from blood samples of HC collected prior to the first probiotic supplementation and after two weeks. At both time points, CD4+CD25+ cells were isolated from freshly isolated PBMCs using the Regulatory T Cell Isolation Kit human (Miltenyi Biotec) according to the manufacturer's instructions. Non-CD4+ cells were depleted and the remaining CD4+ T cells were separated by their CD25 expression, yielding CD4+CD25+ regulatory T cells (Treg) and CD4+CD25− responder T cells (Tresp). We assessed the purity of the isolated Treg and Tresp populations by flow cytometry specific for CD4 and CD25 expression (antibodies are listed in Table 2). CD4+CD25− Tresp cells were labelled with e450 proliferation dye (eBioscience) according to the manufacturers' instructions. The cells were then seeded at different ratios (1:2, 1:4, 1:8, and 1:16) on an anti-CD3 pre-coated (2 μg/mL) 96-well plate together with soluble anti-CD28 (2 μg/mL). As a reference for autologous proliferation of e450 proliferation dye stained cells, Tresp were seeded separately without coculture. Suppression capacity was analysed 72 h later by investigating proliferating CD4+CD25− cells *via* flow cytometry whereby dead cells were excluded by Fixable Viability Dye eFluor® 780 (eBioscience).

### *In vitro* T cell differentiation assay

CD4+CD25−CD45RA+ cells were isolated from freshly isolated PBMCs from healthy donors using the CD4+CD25+CD45RA+ Regulatory T Cell Isolation Kit human (Miltenyi Biotec) according to the manufacturer's instructions. 1 × 10^5^ CD4+CD25−CD45RA+ T cells were cultured on 96-well plates together with T cell TransAct (1:100; Miltenyi Biotec) in the presence of solvent (0.5% ethanol) or indole-3-acetate at different concentrations. After 6 days, the cells were analysed for the frequency of IL-10+ cells in CD4+ viable lymphocytes with flow cytometry. For RNA expression analysis and IL-10 measurements, 2 × 10^5^ CD4+CD25−CD45RA+ cells were cultured in 24-well plates as described above. Cell culture supernatants were harvested for ELISA and cells were washed with PBS prior to RNA isolation. In some experiments, the AHR inhibitor CH-223191 (MedChem Express) was added to the cell culture at a concentration of 5 μM.

### Fecal sample acquisition, handling, and storage

Study participants were asked to provide stool samples prior to probiotic intake, two and six weeks into probiotic intake, and six weeks after the last probiotic intake. One part of the stool sample was immediately stored at −80 °C for future experiments. Another part of the stool sample was directly dissolved in RNA/DNA-shield buffer (Pangea Laboratory licenced from Zymo Research and the Institute of Clinical Microbiology and Hygiene at the University Hospital Regensburg) and stored in a cooling chamber at 4 °C.

### Gut microbiome analysis–DNA extraction and 16S rRNA gene sequencing

Microbiome analysis was performed on stool samples from HC and pwMS at baseline and following a two-week probiotic intervention. Microbial DNA was isolated from 25 mg of frozen stool stored at −80 °C. The extraction process involved Lysing Matrix Y beads (MP Biomedicals, USA) and mechanical disruption using a TissueLyzer II (Qiagen, Germany). Purification of nucleic acids was completed with the MagNA Pure 96 system (Roche Diagnostics, Switzerland). Bacterial 16S rRNA gene copy numbers were quantified by quantitative PCR on a LightCycler 480 II (Roche Diagnostics) using universal primers (764F and 907R) and SYBR Green I Master mix (Roche Diagnostics). Full-length 16S rRNA gene standards were created by cloning PCR products (27F/1492R) amplified from stool DNA of a healthy human donor into the pGEM-T Easy vector (Thermo Fisher Scientific, USA). Sequencing adhered to a ISO 15189 accredited workflow, targeting the V3-V4 hypervariable regions with primers 341F/815R, and DNA was normalised to 1e7 16S rRNA gene copies per sample. Barcoded amplicons were pooled and purified with MagSi-NGSPREP-PLUS beads (Steinbrenner Laborsysteme, Germany) at a 1:1.2 DNA-to-bead ratio. Sequencing library quantification was carried out using the Ion Library TaqMan™ Quantitation Kit. Normalised an pooled library was loaded on a IonChef Instrument and reamplified with the Ion 520™ & Ion 530™ ExT Kit-Chef. Sequencing was performed on the Ion GeneStudio S5 Plus platform (Thermo Fisher Scientific).

### Gut microbiome analysis–data processing and statistical analysis

Raw sequencing data generated from Torrent Suite 5.18 were initially processed using cutadapt 4.4^37^ to remove adaptors and primers, followed by demultiplexing. Reads were trimmed with Trimmomatic 0.39^38^ using a sliding window approach (10-base segments, quality cutoff of 15), discarding reads shorter than 250 bp. Further filtering with vsearch 2.28.1^39^ excluded reads exceeding five expected errors. Zero-radius operational taxonomic units (zOTUs) were defined with an alpha of 2 and a minimum read count of five. Chimeras were identified and removed using uchime3_denovo, and sequences with ≥98% similarity were aligned to the zOTUs *via* usearch_global. Taxonomic classification was performed in R (version 4.4.0) using the IDTAXA classifier from the DECIPHER package (version 2.26.0),^40^ with the Genome Taxonomy Database (GTDB release 220.0,^41^) serving as the reference, applying a bootstrap confidence of 98% and a score threshold of 40.

Alpha diversity was assessed by summarising per sample zOTUs (richness) using the mia package (version 1.1.7) in R. Differences between cohorts were analysed using a Wilcoxon rank-sum test at baseline and after two weeks of treatment. p-Values were adjusted for multiple comparisons using the Benjamini–Hochberg method. Statistical significance of treatment groups was assessed by post-hoc analysis with emmeans::emmeans (version 1.8.4-1). Beta diversity was assessed by calculating BrayCurtis dissimilarities followed by principal coordinates analysis using the mia package (version 1.1.7). Statistical significance between groups was assessed by Permutational Multivariate Analysis of Variance (PERMANOVA) with pairwiseAdonis:pairwise.adonis. Differential abundance analysis between HC and pwMS at baseline and after two weeks of probiotic supplementation was employed by MicrobiomeStat::LinDA (version 1.1,^42^), focussing on features with ≥0.1 percent mean abundance and ≥1 percent prevalence, with statistical significance set at an alpha level <0.1 and a log2-fold cutoff of 1 for zOTUs summarised at the genus level. LinDA analysis was based on a linear mixed-effects model, with treatment groups, timepoints, and their interaction as fixed effects, and individual participants as random effects to account for repeated measures. All plots were generated with ggplot2 version 3.5.1.

### Quantification of short-chain fatty acids in stool samples

Faecal samples for metabolite analysis were processed as recently described.^43^ Briefly, faecal homogenate was prepared by addition of 70% 2-propanol and homogenisation in a gentleMACS dissociator (Miltenyi Biotec, Bergisch Gladbach, Germany). The homogenate was diluted to a final concentration of 2.0 mg dry weight (dw) per mL. Short chain fatty acids were quantified by LC-MS/MS as previously described in detail.^44^

### Quantification of tryptophan metabolites by HPLC−MS/MS

Tryptophan and its downstream metabolites were quantified in stool sample extracts (70% 2-propanol, mass concentration of dried stool: 2 mg/mL (w/v)) and serum samples. The sample preparation protocol was applied as described previously, if not stated otherwise.^45^ Shortly, 10 μL of an aqueous stable isotope-labelled internal standard mixture containing ^13^C_6_-anthranilic acid, ^13^C_7_-3-hydroxyanthranilic acid, ^2^H_5_-5-hydroxyindoleacetic acid, ^13^C_2_-indole-3-acetic acid, ^13^C_10_-kynurenic acid, ^13^C_10_-kynurenine, ^2^H_4_-nicotinamide, ^2^H_4_-nicotinic acid, ^2^H_4_-serotonin, ^2^H_4_-tryptamine, ^2^H_3_-l-DOPA, ^13^C_2_-^15^N_1_-3-hydroxykynurenine, ^2^H_4_-xanthurenic acid, ^2^H_2_-3-indolepropionic acid, and ^2^H-indolelactic acid (10 μM each), as well as ^13^C_11_-tryptophan (200 μM) and ^2^H_3_-quinolinic acid (100 μM) was added to 50 μL of serum and 300 μL stool sample extract. Then, the serum samples were diluted at a ratio of 1:5 with cold 100% MeOH, vortexed, and incubated at −80 °C for 24 h to ensure complete protein precipitation. Serum samples were centrifuged at 10,000×*g* at 4 °C for 5 min, the supernatant was collected. The protein pellets were washed twice with 200 μL of cold 80% MeOH. After centrifugation at 10,000×*g* at 4 °C for 5 min both supernatants of the washing steps were combined with the first supernatant. The collected supernatants were dried using a vacuum evaporator (CombiDancer, Hettich AG, Bäch, Switzerland). A total of 300 μL of stool extracts were dried per sample. All samples were reconstituted in 100 μL of 0.1% formic acid in water.

An ExionLC-30AD HPLC system (AB Sciex, Germany, Darmstadt) was used for the chromatographic separation of target metabolites on an ACQUITY Premier HSS T3, 1.8 μm, 2.1 × 150 mm column (Waters, Germany, Eschborn) reversed-phase column. Gradient elution was performed with mobile phase A consisting of 0.1% formic acid in water and mobile phase B of 0.1% formic acid in acetonitrile. Metabolite detection was performed with a Triple Quad6500^+^ (AB Sciex, Germany, Darmstadt). Peak integration and data evaluation were performed using SciexOS-MQ Software (Version 2.1.6, AB Sciex, Germany, Darmstadt). Results were normalised to serum volume or mass concentration of dried stool, accordingly.

### Statistical analysis

Databases were created in Excel 2013 (Microsoft) and analysed *via* integrated Excel tools, IBM SPSS Statistics 25 (IBM), and GraphPad Prism version 10 (GraphPad Software). Normal distribution was tested by Kolmogorov–Smirnov and Shapiro–Wilk tests. T-tests were applied for normally distributed variables, while non-parametric tests such as Wilcoxon signed-rank test for timepoint comparisons and Mann–Whitney-U for group comparisons were used to analyse the others. Comparisons between more than two groups were analysed using Kruskal–Wallis or 2-way ANOVA, followed by multiple comparisons using Tukey or Šidák's adjusted tests. Data on gut microbiota were analysed by Wilcoxon rank-sum test at baseline and after 2 weeks of treatment. p-Values were corrected for multiple comparisons by the Benjamini and Hochberg method. Data are presented as mean ± SEM; ∗p < 0.05, ∗∗p < 0.01, or ∗∗∗p < 0.001 were considered to be statistically significant. Investigators were blinded to group allocation during data collection and analyses to minimise any potential bias.

### Role of funders

No funding source was involved in the study design, data collection, data analyses, interpretation, writing of this manuscript or the decision to submit for publication.

## Results

### Study design and baseline characteristics

**Fig. 1:**
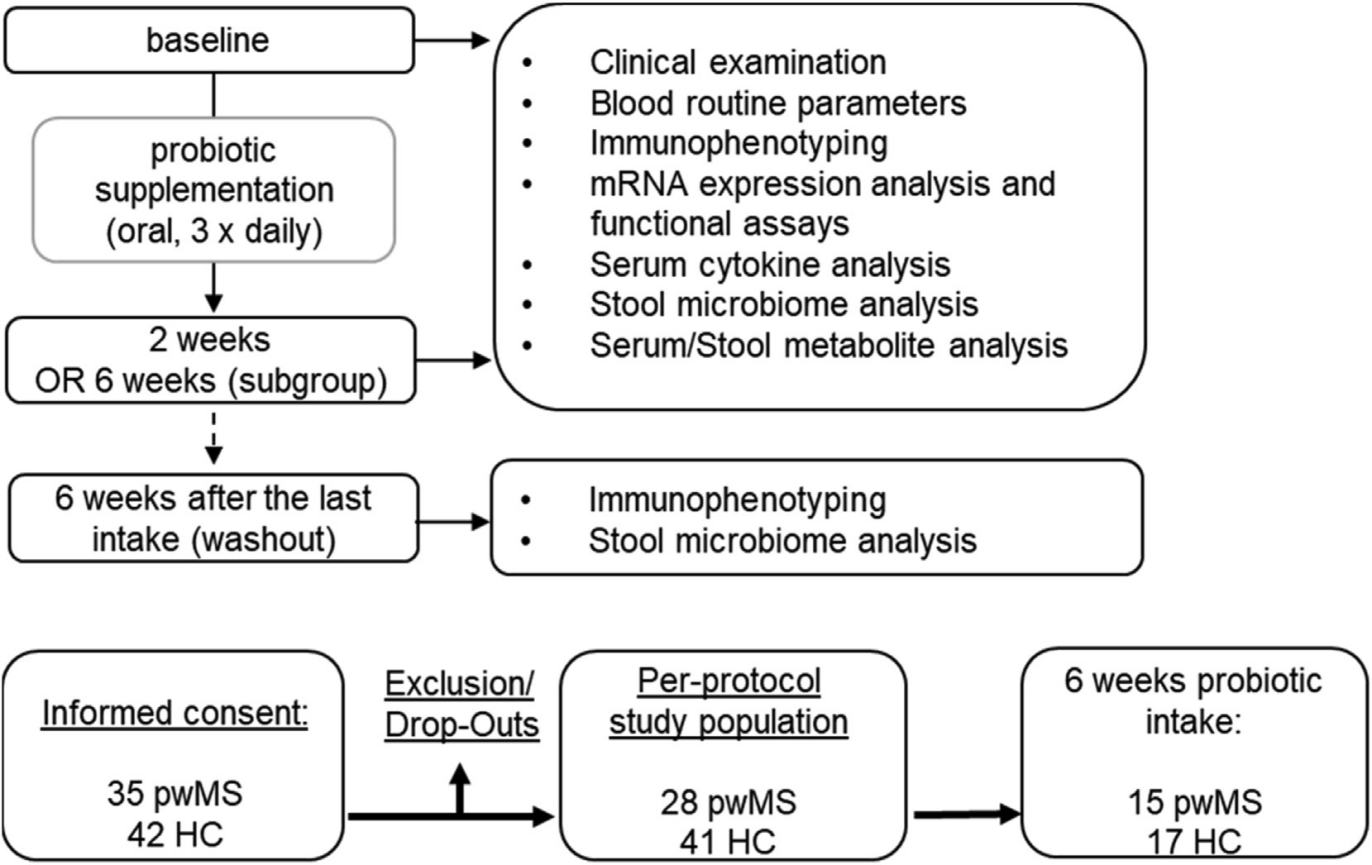
**Study design and baseline characteristics.** This exploratory study investigated immunomodulatory effects of a lactobacilli-containing oral probiotic (Vivomixx®) in a per-protocol study population consisting of healthy controls (HC, n = 41) and people with MS (pwMS, n = 28). All study participants supplemented the probiotic for at least two weeks, a subgroup of 15 pwMS and 17 HC continued the probiotic intake for up to six weeks. PwMS supplemented the probiotic to their existing immunomodulatory therapy. Parameters were analysed before probiotic intervention (baseline) and after two or six weeks of daily probiotic intake and 6 weeks after termination of probiotic supplementation.

Twenty-eight pwMS (46% female, median age 45 (21–64) years, 78% RRMS, median disease duration 12 (1–33) years, median EDSS 3 (1–7)) and 41 HC (63% female, median age 23 (21–63); demographic details in Table 1) were asked to ingest the lactobacilli-rich probiotic named Vivomixx® for 2 weeks. PwMS supplemented the probiotic to their existing immunomodulatory therapy (82% B cell depleted). All participants underwent a thorough medical and neurological examination before the start and at the end of the study. We collected stool samples before the first probiotic intake and after two weeks for microbiome analysis and metabolite measurements. Whole blood was collected at both time points for blood routine measurements, in-depth immunophenotyping, mRNA expression analysis on isolated peripheral blood mononuclear cells (PBMCs) and functional assays as well as serum cytokine and metabolite measurements (Fig. 1). A subgroup of n = 15 pwMS (27% female, median age 48 (31–64) years, 75% RRMS, median disease duration 10 (1–27) years, median EDSS 3 (0–6)) and n = 17 HC (59% female, median age 27 (21–63); demographic details in Table 1) continued the probiotic intake for four more weeks (six weeks of probiotic intake) for immune cell phenotyping and gut microbiome analysis. Stool and blood samples were additionally investigated six weeks after the last probiotic intake (washout).

### Probiotic supplementation shifts the peripheral T cell composition towards an anti-inflammatory phenotype in HC and pwMS

**Fig. 2:**
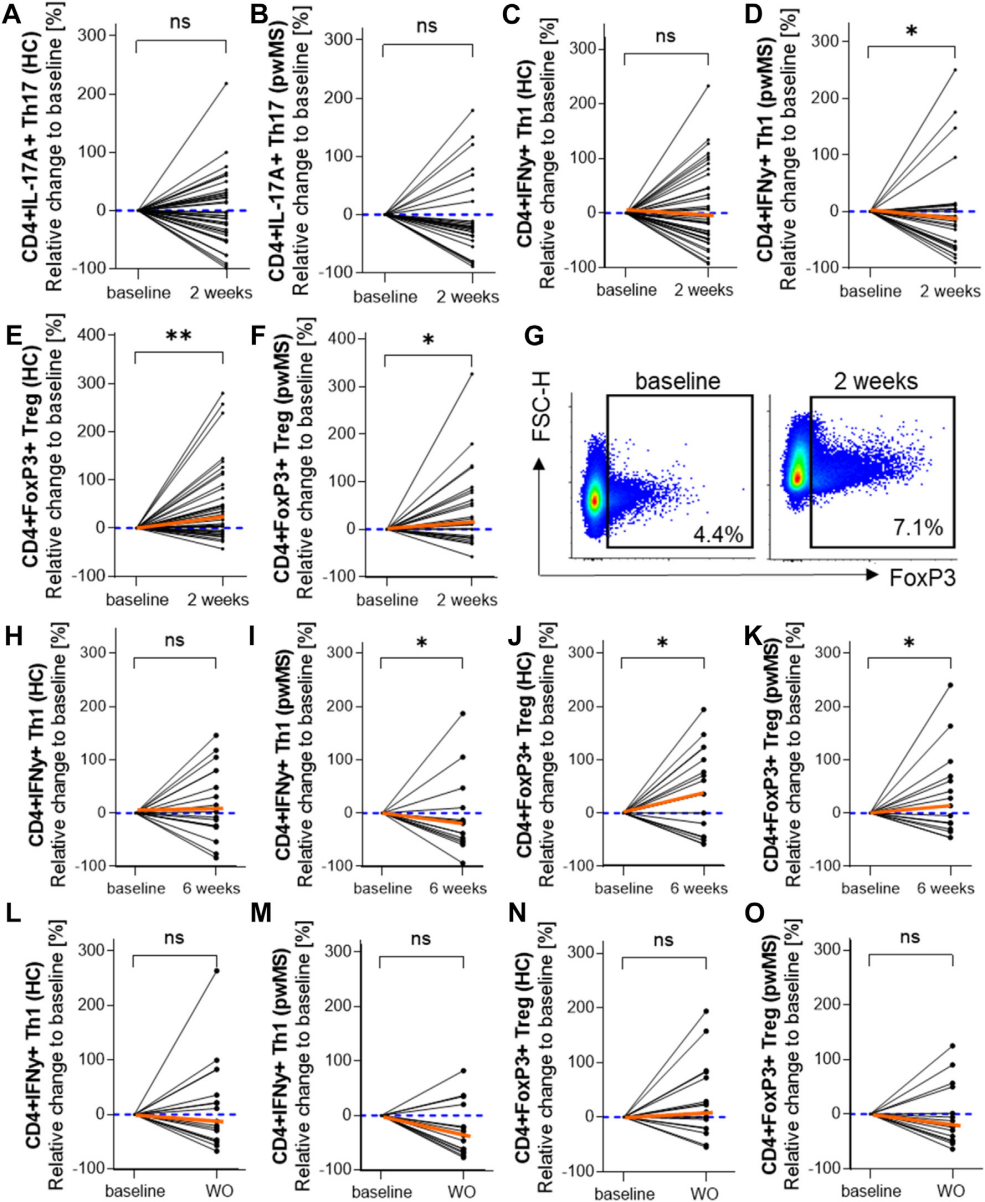
**Probiotic supplementation shifts the peripheral T cell composition towards an anti-inflammatory phenotype in HC and pwMS.** PBMCs from HC and pwMS were analysed for their respective marker expression *via* flow cytometry before (baseline), after two and six weeks of probiotic supplementation and six weeks after termination of probiotic intake (WO). The graphs show relative changes of the analysed cell population normalised to each individual's baseline. Black lines represent individual study participants. Orange lines indicate the median change. (A, B) Two weeks of probiotic intake did not significantly change IL-17A+ Th17 cells in HC (A) and pwMS (B). (C, D) IFNγ+ Th1 cells revealed no effect in HC (C) but a decrease in pwMS (D) after two weeks. (E–G) FoxP3+ Treg cells increased in HC (E) and pwMS (F). (G) Representative dot plots of FoxP3+ Treg cells demonstrate the increase after two weeks of probiotic intake (right) compared to baseline (left). (H, I) Six weeks of probiotic intake did not change significantly Th1 cell frequencies in HC (H) but decreased Th1 cells in pwMS (I). (J, K) Treg cell frequencies were significantly increased in HC (J) and pwMS (K) after six weeks. (L–O) Th1 and Treg cell frequencies were analysed 6 weeks after the last probiotic intake (washout, WO). Relative frequencies of Th1 cells reduced in HC (L) and pwMS (M), whereas Treg cell frequencies remained unchanged in HC (N) but dropped in pwMS (O) compared to baseline frequencies. Significance for changes over time were calculated using unnormalised cell frequencies with Wilcoxon matched-pairs signed rank test. (A) n = 41, p = 0.672. (B) n = 28, p = 0.333. (C) n = 40, p = 0.952. (D) n = 28, ∗p = 0.029. (E) n = 37, ∗∗p = 0.005. (F) n = 28, ∗p = 0.036. (H) n = 17, p = 0.963. (I) n = 15, ∗p = 0.0302. (J) n = 17, ∗p = 0.0295. (K) n = 15, ∗p = 0.0129. (L) n = 17, p = 0.404. (M) n = 15, p = 0.064. (N) n = 17, p = 0.382. (O) n = 15, p = 0.629.

Routine blood tests revealed no significant changes in pwMS and HC after two and six weeks of probiotic intake compared to baseline parameters (Suppl. Table S1). Flow cytometry analysis of blood immune cells in pwMS and HC before the first probiotic intake and after two or six weeks revealed no effect on B cells (CD19+), natural killer (NK) cells (CD56brightCD16dim, CD56brightCD16−, and CD56dimCD16bright), monocytes (classical CD14++CD16−, non-classical CD14+CD16++, intermediate CD14++CD16+), and CD4+/CD8+ effector or memory T cells (Suppl. Table S2). Additional analysis of intracellular markers in CD4+ T cells revealed a potential shift towards an anti-inflammatory phenotype under probiotic intake (Fig. 2). Although 54% of HC and 71% of pwMS showed decreased CD4^+^IL-17A^+^ cell frequencies after two weeks of probiotic consumption compared to their baseline levels (Fig. 2A and B), the mean change in Th17 cell frequencies did not reach statistical significance in both groups. In contrast, probiotic intake was parallelled by a significant decrease in the number of CD4^+^IFNγ^+^ Th1 cells in pwMS (Fig. 2D) but not in HC (Fig. 2C). Two-thirds of pwMS showed decreased Th1 cells with a mean reduction of 24% (Suppl. Table S2). Moreover, 71% of pwMS responded with increased FoxP3^+^ Treg cell frequencies after two weeks of probiotic intake (Fig. 2F and G). The mean frequencies of Treg cells increased by 44% (Suppl. Table S1). In HC, we detected a similar increase in Treg cell frequencies in 63% of the study group (Fig. 2E). The effect of probiotic intake on peripheral Th1 and Treg cells persisted over 6 weeks of intake in both HC and pwMS (Fig. 2H–K). HC displayed no significant change in the mean frequencies of Th1 cells after 6 six weeks of probiotic intake (Fig. 2H), whereas pwMS responded with significantly decreased Th1 cell frequencies (Fig. 2I). Treg cells were increased in both, HC (Fig. 2J) and pwMS (Fig. 2K). During the washout period, Th1 cell levels remained at lower levels in HC and pwMS compared to their baseline levels (Fig. 2L and M), whereas the effect of probiotic supplementation on Treg cell frequencies could not persist after the termination of probiotic intake (Fig. 2N and O).

### Probiotic supplementation increases IL-10 production and the suppressive capacity of Treg cells

**Fig. 3:**
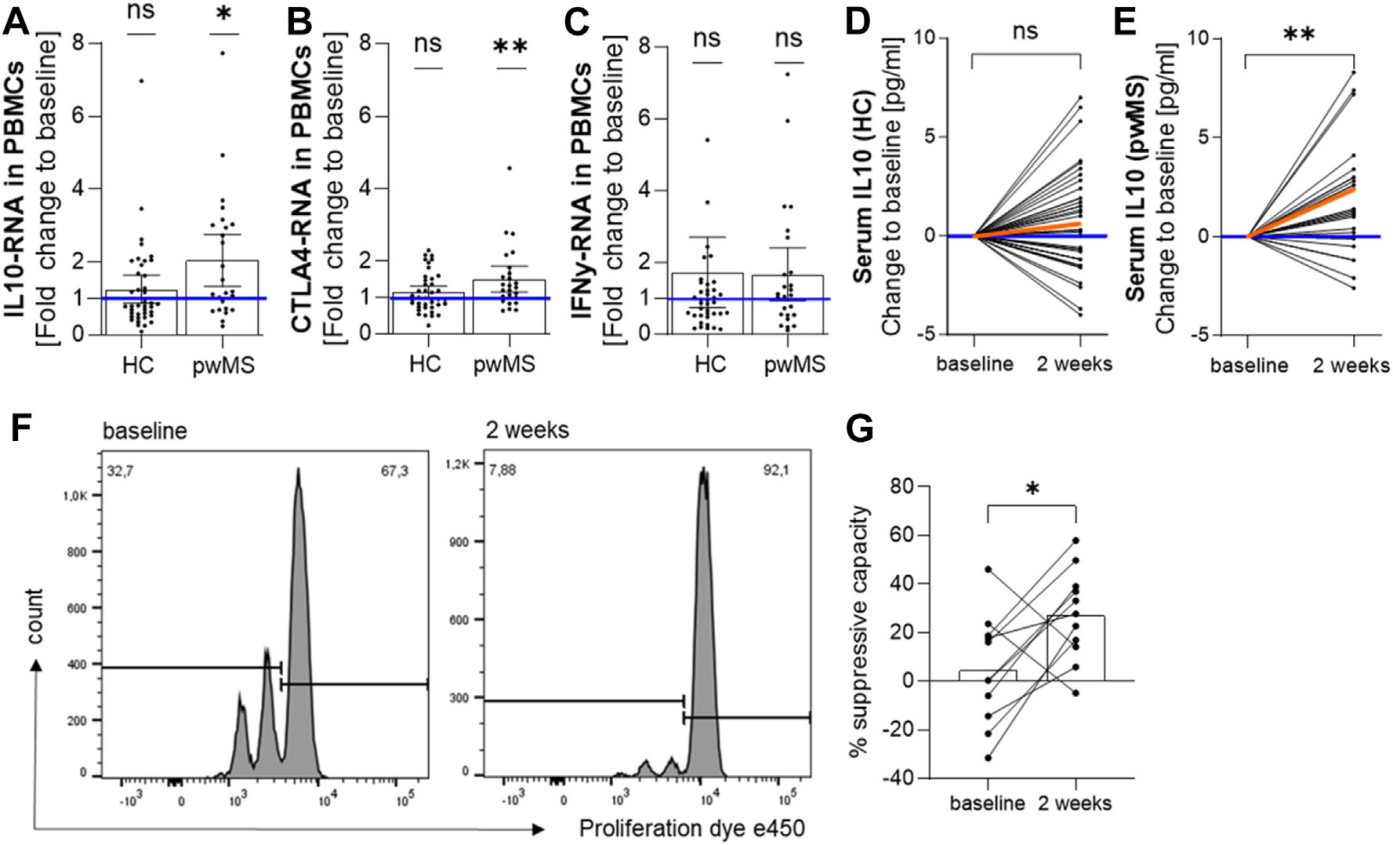
**Probiotic supplementation increases IL-10 production and the suppressive capacity of Treg cells.** (A–C) mRNA expression analysis in PBMCs of HC and pwMS was analysed at baseline and after two weeks of probiotic intake. (A) *IL10* gene expression significantly increased in pwMS after two weeks of probiotic supplementation but not in HC. (B) *CTLA4* gene expression significantly increased in pwMS after two weeks of probiotic supplementation but not in HC. (C) *IFNG* gene expression remained unchanged in pwMS and HC. Graphs show individuals as points and mean ± 95% CI. (D, E) The concentration of IL-10 was analysed by ELISA in serum samples of HC (D) and pwMS (E) at baseline and after two weeks of probiotic intake. The IL-10 concentration increased in pwMS but not in HC. Graphs show relative changes normalised to each individuals' baseline. Black lines represent individual study participants. The orange line indicates the mean change. (F) Representative histograms of the *in vitro* suppression assay show the proliferation of CD4+ PBMCs isolated from HC before (left) and after 2 weeks of probiotic intake (right). (G) Treg cell suppressive capacity in HC increased after two weeks of probiotic supplementation. Significance for changes over time was calculated using unnormalised values with Wilcoxon matched-pairs signed rank test in A–G. (A) HC: n = 40, p = 0.727; pwMS: n = 25, ∗p = 0.019. (B) HC: n = 40, p = 0.332; pwMS: n = 25, ∗∗p = 0.004. (C) HC: n = 40, p = 0.793; pwMS: n = 25, p = 0.353. (D) n = 40, p = 0.179. (E) n = 28, ∗∗p = 0.002. (G) n = 11, ∗p = 0.0186.

We further analysed the expression of IL-10 as a signature cytokine of anti-inflammatory Treg cells as well as CTLA-4 as an important immune checkpoint to downregulate immune responses. PwMS responded with increased *IL10* (2.0-fold ± 1.7; Fig. 3A) and increased *CTLA4* (1.5-fold ± 0.9; Fig. 3B) mRNA expression in isolated PBMCs after probiotic intake whereas we observed no effect in HC. *IFNG* expression representative for Th1 cells changed in neither group (Fig. 3C). Additional cytokine measurements in serum samples revealed a significantly increased concentration of IL-10 in pwMS after the probiotic intake compared to baseline (Fig. 3E). This effect could not be detected in HC (Fig. 3D). Moreover, we did not identify any changes in the concentration of other T cell or monocyte related cytokines such as IL-17, IFNγ, IL-23 or IL-6 (Suppl. Table S2). The increase of Treg cells concomitant with the enhanced IL-10 levels prompted us to investigate a potential effect of probiotic intake on Treg functionality. Therefore, Treg cells were isolated from HC prior to probiotic intake and after two weeks and co-cultured with T responder (Tresp) cells from the same donor to investigate the efficiency of Treg cells to suppress Tresp proliferation. We detected a significantly increased suppressive capacity of Treg cells after probiotic intake (Fig. 3F and G).

### Probiotic supplementation increases indole-3-acetic acid (IAA) in stool and serum and IAA enhances IL-10 production in T cells *in vitro*

**Fig. 4:**
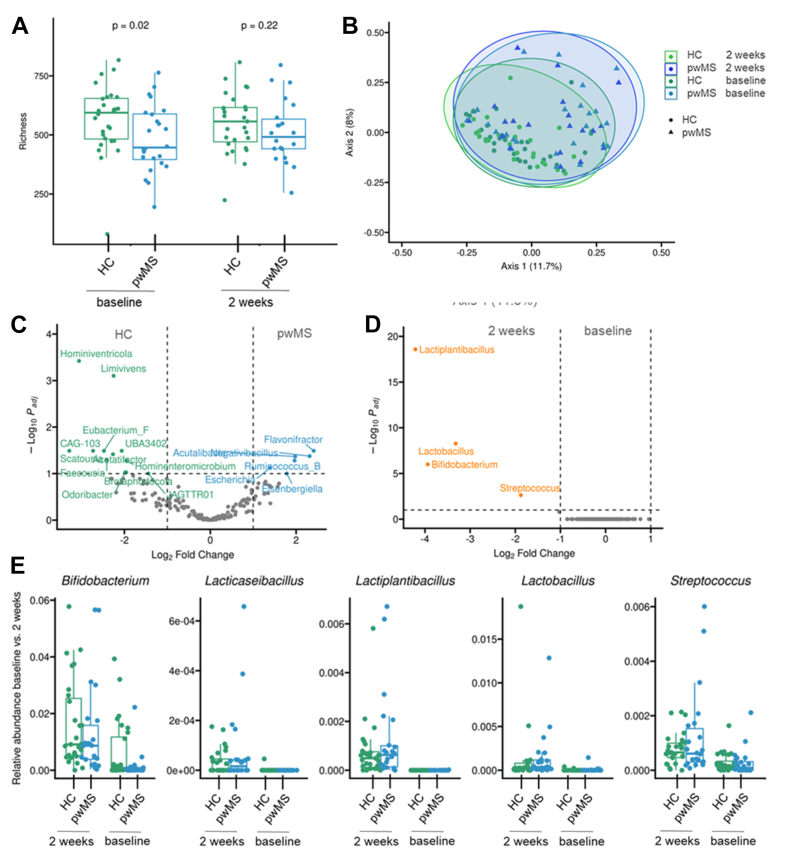
**Probiotic bacterial strains efficiently colonise the gut microbiome of HC and pwMS.** 16S rRNA sequencing was performed in stool samples from HC and pwMS before (baseline) and after two weeks of probiotic supplementation (HC n = 27 per timepoint; pwMS n = 22 per timepoint). (A) Alpha diversity of the gut microbiome in pwMS (green) and HC (blue) revealed a higher microbiota richness in HC compared to pwMS but no effect due to probiotic supplementation. (B) Beta diversity as presented by Bray–Curtis permutational multivariate analysis of variance differed between HC and pwMS, but it was not affected by probiotic intake. (C) Differential abundance analysis with the Linear Model for Differential Abundance Analysis of High-dimensional Compositional Data (LinDA) revealed a differential abundance of bacterial genera between HC and pwMS at baseline. (D) Differential abundance analysis with LinDA confirmed a successful colonisation of all probiotic bacteria in the gut microbiota after two weeks of probiotic supplementation. (E) The enrichment of all probiotic bacteria could be confirmed in both study groups (green: HC, blue: pwMS; left: 2 weeks of probiotic intake, right: baseline). Differences between cohorts were analysed using a Wilcoxon rank-sum test at baseline and after two weeks of treatment. p-Values were adjusted for multiple comparisons using the Benjamini–Hochberg method.

**Fig. 5:**
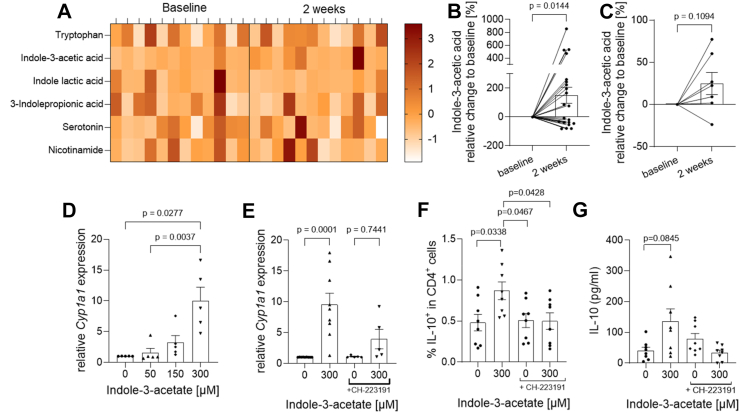
**Probiotic supplementation increases indole-3-acetic acid (IAA) in stool and serum and IAA enhances IL-10 production in T cells *in vitro*.** (A) Heat-map of microbial metabolites analysed in stool samples of pwMS before probiotic supplementation (baseline) and after two weeks by high performance liquid-chromatography–mass spectrometry. (B, C) Concentrations of IAA in (B) stool samples and (C) serum samples of pwMS before probiotic intake (baseline) and after two weeks. Black lines represent individual pwMS. The graphs show relative changes normalised to each individual's baseline. Wilcoxon matched-pairs signed rank test B: n = 22, p = 0.0460; C: n = 7, p = 0.1094. (D) Naïve CD4+ T cells from HC were cultured under co-stimulatory anti-CD3/anti-CD28 conditions in the presence or absence of different concentrations of IAA. IAA increased *CYP1a1* gene expression in *in vitro* cultured T cells (n = 5 per group; Kruskal–Wallis test with post-hoc Dunn's test and Bonferroni adjustment for multiple comparisons, p = 0.0277; p = 0.0037). (E–G) Naïve CD4+ T cells from HC were cultured under co-stimulatory anti-CD3/anti-CD28 conditions in the presence or absence of 300 μM IAA with or without 5 μM of the AHR inhibitor CH-223191. (E) The IAA induced increase in *CYP1a1* gene expression was prevented by simultaneous addition of CH-223191 (n = 5 per group). (F) Flow cytometry analysis of IL-10+ cells among CD4+ T cells revealed a relative increase under IAA and no effect upon additional treatment with CH-223191 (n = 8 per group; Two-way ANOVA with Šidák's Multiple comparisons test, factor 1: IAA concentration; factor 2: CH-223191 addition). (G) IL-10 concentrations were analysed in cell culture supernatants by ELISA and revealed a significant increase in IAA treated T cells that was blocked by the simultaneous addition of CH-223191. (n = 8 per group; Two-way ANOVA with Šidák's Multiple comparisons test; factor 1: IAA concentration; factor 2: CH-223191 addition).

To elaborate whether the immune modulatory effects of probiotic supplementation are linked to changes in the gut, we next investigated the microbiota composition and metabolite production before and after probiotic intake. We observed a significant difference in alpha and beta diversity (Fig. 4A and B) of the gut microbiome between HC and pwMS at baseline, with a lower bacterial richness in pwMS compared to HC. Yet, probiotic intake did not affect the microbial diversity in both groups (Fig. 4A and B). Coinciding with previous findings, we detected a higher abundance of *Flavonifractor* and *Escherichia*^25^ but a lower abundance of SCFA producers such as *Odoribacter* in pwMS (Fig. 4C). These differences did not affect the efficient colonisation of probiotic bacterial strains in the gut microbiota of pwMS compared to HC. We detected an increase of all probiotic bacterial strains in both groups already after two weeks of Vivomixx® intake (Fig. 4D and E). The bacterial strains showed a similar relative abundance after 6 weeks of probiotic intake and, interestingly, dropped to baseline levels after the termination of probiotic intake (Suppl. Figure S1). We additionally investigated microbial metabolites in feces and serum samples. We investigated SCFAs known to induce functionally competent Treg cells in pwMS,^25^ but observed no changes after two weeks of probiotic intake compared to baseline (Suppl. Figure S2A–D). We additionally investigated metabolites of the tryptophan/kynurenine pathway such as indoles, because we had identified recently their involvement in lactobacilli-mediated amelioration of MOG-EAE.^26^ We detected an increase in fecal concentrations of indole-3-acetic acid (IAA) in pwMS after two weeks of probiotic intake compared to baseline (Fig. 5A and B), but only minor to no effects on indole-3-lactic acid (Suppl. Figure S2E) and other tryptophan-kynurenine metabolites (Suppl. Figure S2F–I), suggesting a specific increase of IAA due to Vivomixx® intake. We also detected a non-significant trend towards increased serum concentrations of IAA in pwMS supplementing the probiotic for two weeks (Fig. 5C). Yet, likely due to the small sample size, this effect was statistically not significant. However, we suspected a link between increased IAA concentrations and the induction of IL-10 producing CD4+ T cells. To investigate this, naive CD4+ T cells from peripheral blood of healthy donors were cultured in the presence of T cell antigen receptor and CD28 signalling without and with addition of IAA (Fig. 5D–G). The addition of IAA resulted in a concentration-dependent induction of *CYP1a1* expression with a 10-fold increase in cells treated with 300 μM IAA (Fig. 5D). These data suggested an activation of aryl hydrocarbon receptor (AHR) signalling in CD4+ T cells. To further investigate this effect, AHR signalling was effectively blocked by culturing IAA-treated cells in the presence of the AHR inhibitor CH-223191 (Fig. 5E). Interestingly, the addition of IAA increased the frequency of IL-10 producing CD4+ T cells (Fig. 5F), but not IFNγ or IL-17A producing CD4+ T cells (Suppl. Figure S3A and B). Pharmacological blockade of the AHR inhibited the effect of IAA on IL-10+CD4+ T cell frequencies (Fig. 5F). Coinciding with this, we detected an increased IL-10 concentration in cell culture supernatants of IAA treated cells but not in cell cultures additionally treated with CH-223191 (Fig. 5G). Additional experiments with naïve CD4+ T cells isolated from pwMS also revealed an AHR-dependant increase of IL-10 producing CD4+ T cells due to IAA addition (Suppl. Figure S3C and D). These data indicate that a probiotic rich in lactobacilli may enhance the formation of AHR agonists in the gut that may directly induce a regulatory phenotype in CD4+ T cells.

## Discussion

Microbiota living in the gut exert immunomodulatory power, but the specifics in MS remain unclear.^46^ Meanwhile, mounting evidence has shown a so called dysbiosis in the gut microbiota of pwMS, including a depletion of lactobacilli.^17^, ^18^, ^19^, ^20^ Therefore, it has been hypothesised that probiotics containing lactobacilli might alleviate inflammatory dysregulation in pwMS, potentially *via* increased SCFA or tryptophan metabolite production.^14^ Our results support this hypothesis, clearly connecting microbial tryptophan metabolites to specific changes in MS patients' immune system after a probiotic intervention.

Lactobacilli have previously been credited with the ability to induce IL-10 producing Treg cells in EAE diseased mice.^47^^,^^48^ Furthermore, oral lactobacilli supplementation in mice has been correlated to relieved EAE,^26^^,^^48^^,^^49^ and favourable effects have been observed in pwMS.^31^^,^^33^ Our study focused on effects in the blood and stool and did not investigate changes to lymphoid or nervous tissues due to the inherently invasive necessities of such research. Our results nonetheless show that an oral probiotic containing lactobacilli increase Treg cells in both, healthy humans and pwMS, indicating Treg cell development.^50^ Yet, numerical changes alone do not signify functional relevance. It has rather been observed that Treg cells in pwMS are dysfunctional, connected to FoxP3 deficiency and a lack of IL-10 production.^13^ Within this study, we found increased IL-10 levels in serum samples of pwMS after probiotic treatment. We furthermore detected increased *IL10* and *CTLA4* RNA expression in patients' PBMCs. Both factors are part of a Treg-inducing feedback-loop.^12^^,^^51^ Yet, we cannot prove which types of PBMCs were responsible for the observed effects. Nevertheless, our results are highly indicative of a strengthened Treg-mediated immunoregulation. We performed *in vitro* suppression assays to verify whether the observed Treg increase coinciding with enhanced IL-10 levels would suppress a modelled (auto-) immune response. As expected, Treg isolated from HC were able to suppress the proliferation of stimulated CD4+ cells from the same donors, and an increased suppressive capacity could be detected after lactobacilli-enriched probiotic supplementation. Whether the findings are transferable to Treg cells isolated from pwMS remains to be proven. However, the increased number of IL-10 producing Treg cells in pwMS coinciding with a significant and tentative decrease in inflammatory Th1 and Th17 cell frequencies, respectively, suggest an enhancement of Treg functionality after probiotic treatment also in pwMS. A recent study including 30 pwMS supports our results, as supernatants from cultures containing lactobacilli were able to decrease Th1 cell differentiation in patients' CD4+ cells *in vitro*.^52^ The study did not find changes in Th17 differentiation, but IL-17 secretion was nonetheless decreased significantly.^52^ In MS, Th1 and Th17 cells are widely associated with disease activity, and the observed numerical decrease might therefore represent an alleviating shift away from (auto-) inflammation towards a well-regulated state. Other studies investigating the influence of probiotic intake in pwMS observed changes in pro-inflammatory cytokine concentrations^32^^,^^33^ or reduced numbers of inflammatory monocytes.^35^ Here, we did not identify changes in IL-6 or IL-23 levels or altered monocyte frequencies. Yet, the formulation of the investigated probiotics differs between the studies,^53^ and it is likely that a different composition of commensals in the different probiotics might result in different immunological effects. We utilised an existing probiotic formula containing eight different strains for our study. Further research is necessary to untangle the specific effects of each strain and the most beneficial combinations. Moreover, we cannot generally transfer our results to all groups of pwMS. Although this study mainly involved patients with a relapsing-remitting disease course, the immunological consequences of probiotic intake might differ in patients with a chronic disease course. Moreover, our cohort was too small to define differences between therapeutic groups or patients with different disease durations. Most patients in our study had received a B cell depleting therapy (CD20-mononuclear antibody Ocrelizumab), thus limiting our study's generalisability. However, the fact that the probiotic intake can enhance functionally active Treg cells even in immune-depleted pwMS suggests a rather potent immune-modulatory effect. Due to the duration of pre-existing anti-CD20 treatment in our study, we assume that the immune status of the patients is relatively stable and we do not suggest major interferences of the B-cell depleting therapy with the observed changes in Treg and Th1 cell frequencies in pwMS. Yet, future studies would be necessary to prove this. In addition, this is not a multi-centre study and all patients were recruited from a single hospital. Therefore, the data cannot be easily generalised to all patients with MS. The age difference between our two groups could certainly also be discussed as a possible confounder. However, our interest focused on intra-individual changes in individual pwMS or HC over time. Hence, we performed a pre- post-analysis on the effect of probiotic intake in each study participant to identify potential intrapersonal effects rather than comparing the effects between two different groups.

In line with previous reports,^17^, ^18^, ^19^^,^^25^ 16S rRNA sequencing revealed a different microbial composition in pwMS compared to HC, involving an enrichment of some pathobionts (*Flavonifractor* and *Escherichia*) and a reduction of SCFA producing bacteria (*Odoribacter*). Moreover, pwMS but also HC displayed low levels of *Lactobacillus* genera at baseline. A recent study reported that supplementation of a probiotic rich in lactobacilli was associated with improved EDSS,^31^ indicating a potential benefit of gut colonisation by *Lactobacillus* species in pwMS. Here, we detected a rapid increase in gut colonisation by *Lactobacillus* and also *Bifidobacterium* and *Streptococcus species* already after two weeks of probiotic Vivomixx® intake. Whether Vivomixx® supplementation also influences the clinical course of MS remains to be proven in a future study. Of note, the increase in *Lactobacillus* genera induced by the probiotic intake did not persist following discontinuation of Vivomixx®. This implies that probiotic supplementation may have to be continued indefinitely.

We also questioned whether changes to the Treg-IL-10-loop might be rooted in altered microbiota metabolite production. We focused on tryptophan and tryptophan-derived metabolites such as serotonin, kynurenine and different indole derivatives. Although we did not control for eating habits besides exclusion of vegan nutrition, supplementation of vitamins (except Vitamin-D and Vitamin-B12), and previous probiotic intake, we found a specific increase in indole-3-acetate (indole-3-acetic acid, IAA) concentrations in MS patients' blood and stool under probiotic intervention. Recent animal studies have shown that IAA reduced the severity of inflammatory models.^54^^,^^55^ Most notably, IAA upregulated FoxP3, and increased production of IL-10 in inflamed mice.^54^ We found strikingly similar effects in this study. In our human cell cultures, IAA increased both the frequency of CD4+IL-10+ T cells and the concentration of IL-10 in supernatants, which is highly indicative of regulatory function. These combined results clearly suggest a role of increased IAA-production *via* the gut microbiota in our understanding of increased Tregs and IL-10-production in the blood of pwMS. Furthermore, other indole derivatives could be linked to reduced CNS inflammation in the experimental model of MS,^30^ indicating another beneficial involvement during MS pathogenesis. The increase in *CYP1a1* gene expression and the effectiveness of the pharmacological blockade with the AHR-inhibitor CH-223191 in IAA-treated T cells indicates a possible involvement of the AHR-signalling pathway.^56^ Tryptophan metabolites are widely accepted to act as AHR agonists or antagonists with various effects, depending on exact metabolite and systemic circumstances.^57^ IAA improved gut motility *via* AHR activation,^58^ and AHR activation, in turn, has been shown to control immune processes relevant during MS pathology.^59^^,^^60^ Recent data observed a negative correlation of serum AHR agonist levels and disability in patients with RRMS and PMS.^61^ The increase in concentration of selected AHR ligands *via* probiotic modulation may thus represent a promising therapeutic approach. However, future studies will be necessary to prove this.

In summary, our study identified possible future targets for the therapy of neuroinflammatory disease. We were able to link probiotic supplementation to a beneficially modified microbiota metabolite production with the capacity to shift the patient's immune system towards a regulatory phenotype. Overall, since the gut microbiota is currently not the primary therapeutic target for MS, we expect that probiotic modulation in MS may be a complementary approach to the existing baseline disease-modifying therapies.

## Contributors

CT: recruited study participants, conducted experiments, acquired and analysed data, wrote the manuscript. MK: recruited study participants, conducted experiments, acquired and analysed data. DF: recruited study participants and supported data analysis. SHe, KD, PJO: performed gut metabolite measurements and data analysis. GL: performed stool metabolite extractions and SCFA measurements, analysed the data. AH, AG: performed gut microbiota analysis, acquired and analysed the data. DHL, KA: recruited study participants, taught and supervised the clinical students. RAL: conceptualised the project, provided funding, and guidance to all authors involved in data analysis and interpretation. SH: performed data acquisition and analysis, conceptualised and managed the project, provided guidance to all authors involved in data analysis and interpretation, and wrote the manuscript. All authors read and approved the final manuscript. CT, RAL, and SH have directly accessed and verified the underlying data of this study. RAL and SH were responsible for the decision to submit the manuscript.

## Data sharing statement

Source data is provided with this paper. 16S rRNA sequencing data were uploaded to the European Nucleotide Archive (ENA) with the accession number PRJEB85554. Raw data and analyses can be accessed by researchers on request to Stefanie Haase (Stefanie.haase@ukr.de).

## Declaration of interests

The Authors declare that there is no conflict of interest.

## Appendix ASupplementary data

Suppl. Tables and Suppl. Figures
