# Supplementary material for: A probiotic approach identifies a Treg-centred immunoregulation via modulation of gut microbiota metabolites in people with multiple sclerosis and healthy individuals
Source: eBioMedicine. 2025 May 12;116:105743. doi: 10.1016/j.ebiom.2025.105743 (PMC12137156; doi:10.1016/j.ebiom.2025.105743)
Supplement: Suppl. Tables and Suppl. Figures [file mmc1.docx]

**Supplementary Material**

**Supplementary Table 1.** Blood routine parameters in HC and pwMS before the probiotic intake (baseline) and after two and six weeks. Data are given as mean ± SEM.


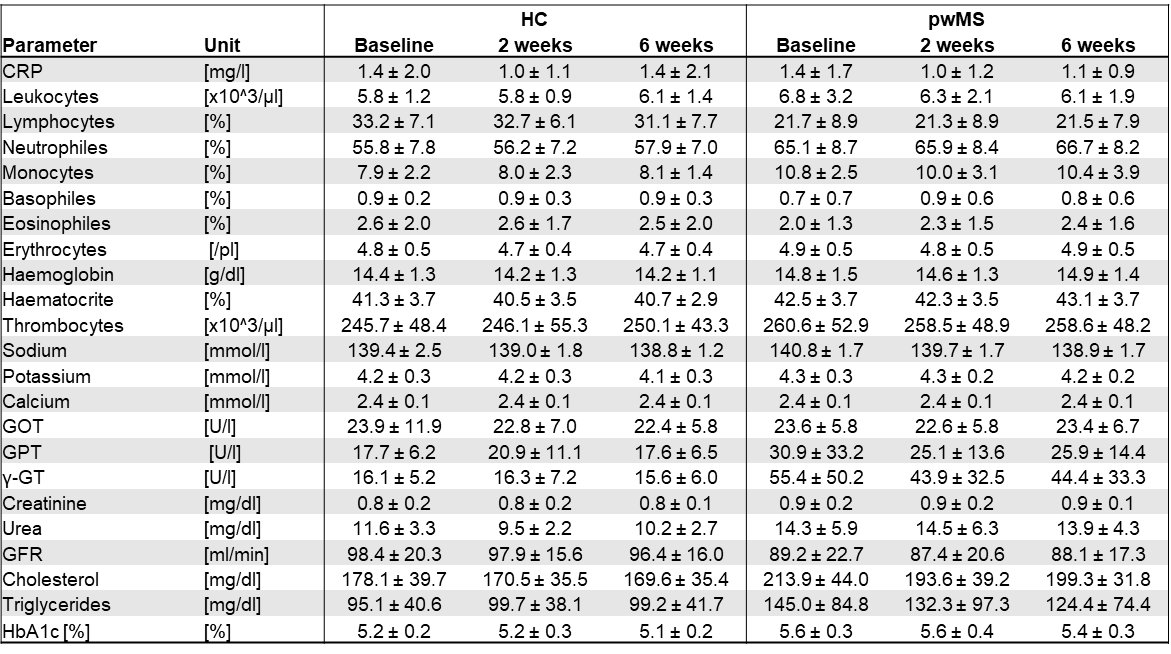


Abbreviations: CRP: C-reactive protein; GOT: glutamate-oxalacetate-transaminase; GPT: glutamate-pyruvate-transaminase; yGT: gamma-glutamyltransferase; GFR: glomerular filtration rate; HbA1c: hemoglobin A1c.

**Supplementary Table 2.** Blood immune cells and serum cytokine concentrations in HC and pwMS at baseline and after two and six weeks of probiotic supplementation. Data are given as mean ± SEM.


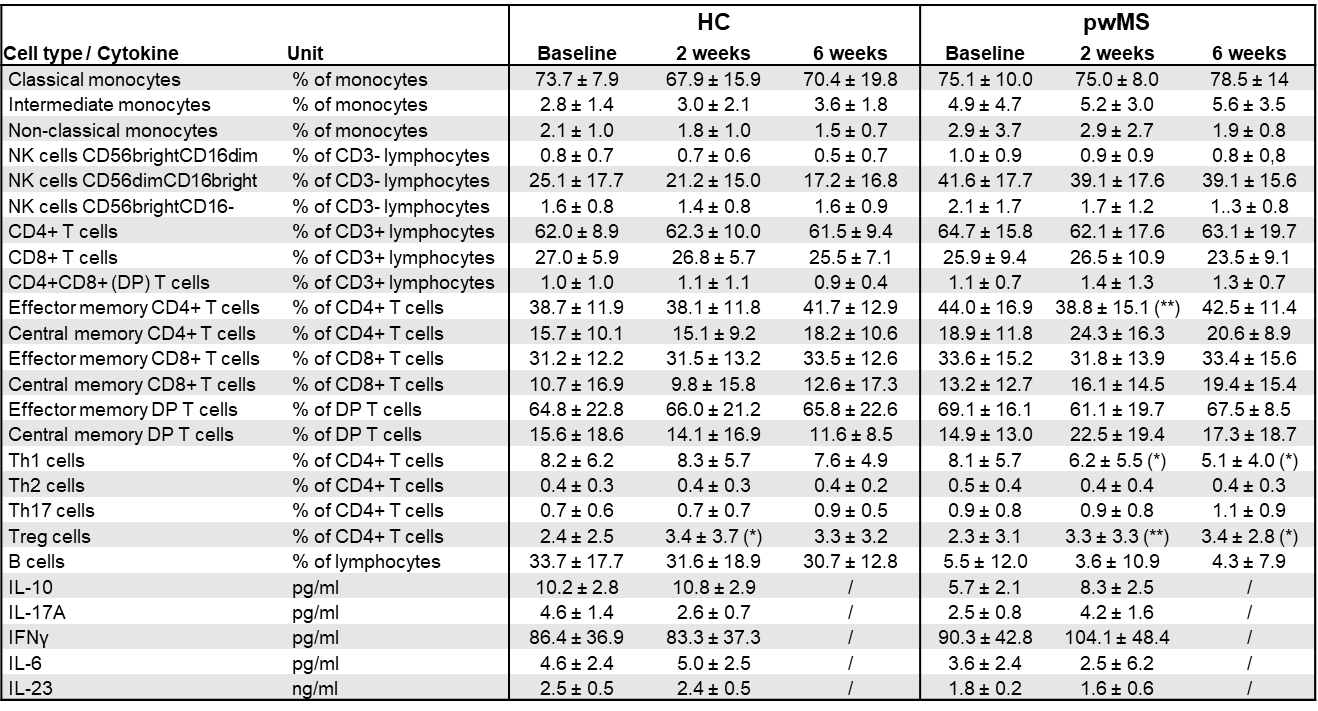


**Supplementary Table 3.** Detailed information on all used reagents.

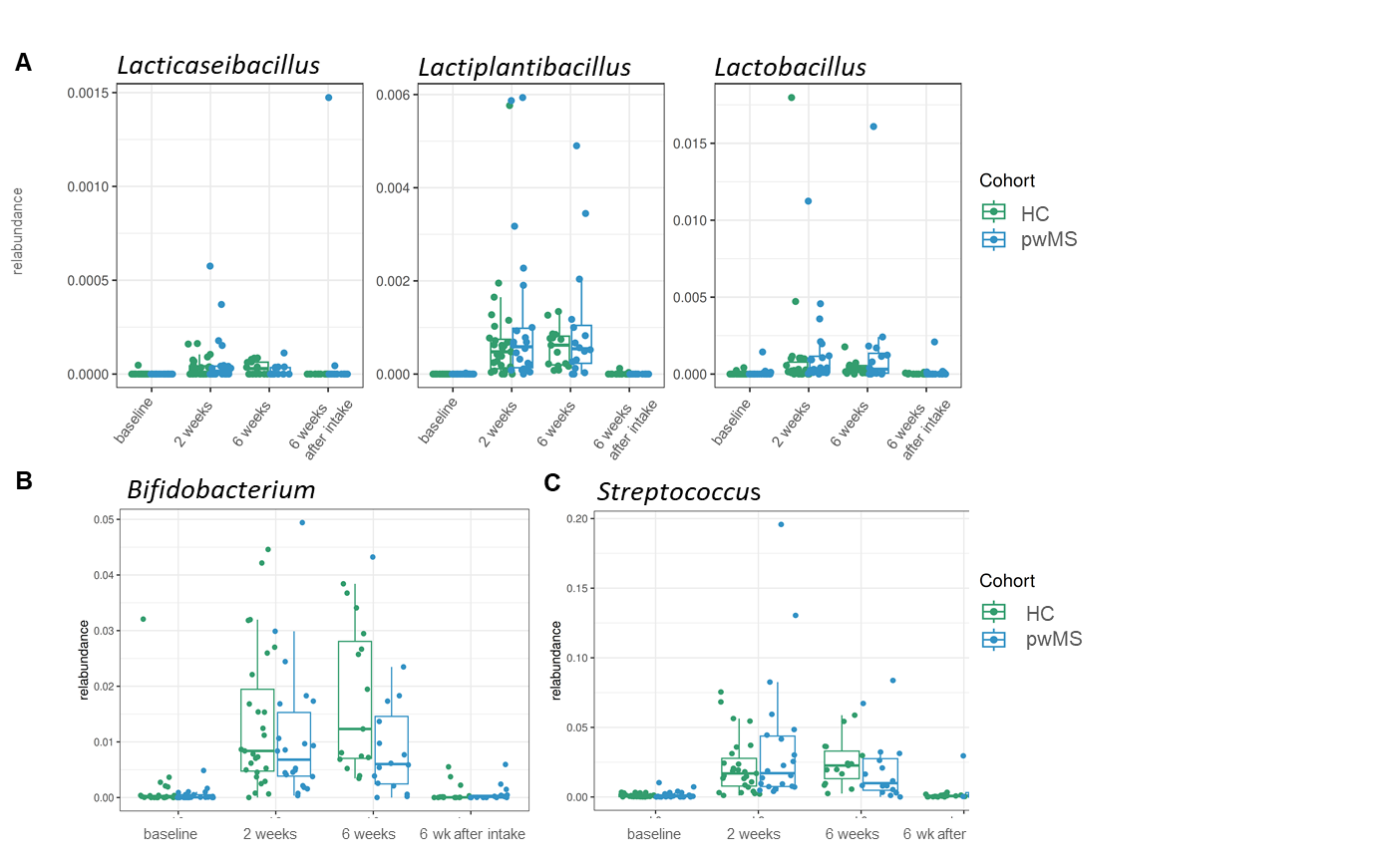


**Supplementary Figure 1. Relative abundance of probiotic bacterial strains remained constant during the probiotic intake but dropped to baseline levels after discontinuation.** Differential abundance analysis with LinDa confirmed a successful colonization of all probiotic bacteria in the gut microbiota after two and six weeks of probiotic supplementation and revealed a drop towards baseline levels six weeks after the last probiotic intake. Baseline HC n= 27, pwMS n=24; 2 weeks HC n=26, pwMS n=22; 6 weeks HC n=15, pwMS n=16; 6 weeks after intake HC n=17, pwMS n=16.


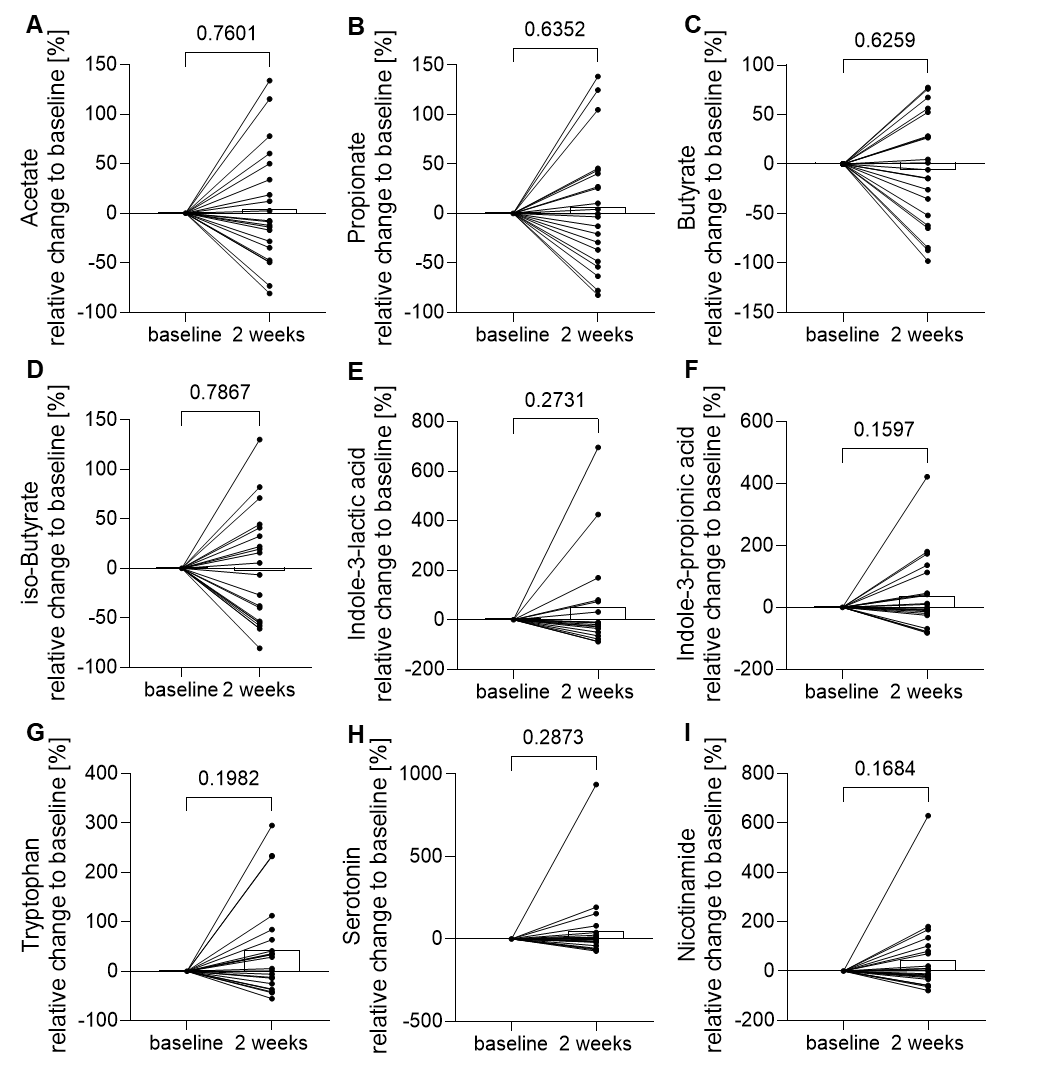


**Supplementary Figure 2.** Microbial metabolites were analyzed in stool samples of pwMS before probiotic supplementation (baseline) and after two weeks by high performance liquid-chromatography–tandem mass spectrometry. Black lines represent individual pwMS. The graphs show relative changes normalized to each individual’s baseline for (A) acetate, (B) propionate, (C) butyrate, (D) iso-butyrate, (E) indole-3-lactate, (F) indole-3-propionate, (G) tryptophan, (H) serotonin, and (I) nicotinamide. Data were analyzed by Wilcoxon matched-pairs signed rank test and revealed no significant changes for any of the analyzed metabolites (n=22 per metabolite).


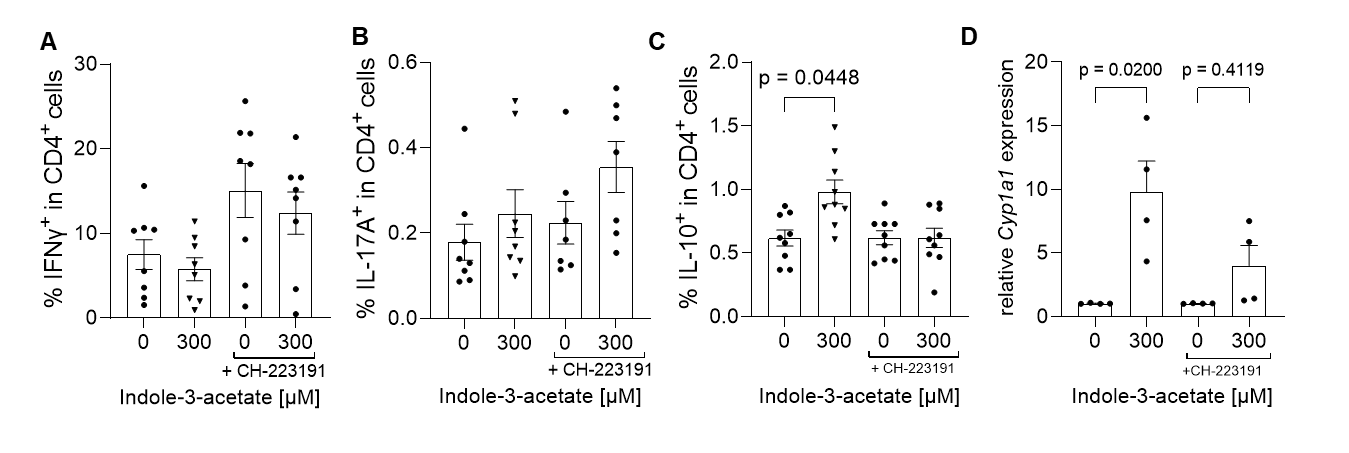


**Supplementary Figure 3. Effects of Indole-3-acetate treatment on human CD4+ T cells in vitro.** (A, B) Naïve CD4+ T cells from HC were cultured under co-stimulatory anti-CD3/anti-CD28 conditions in the presence or absence of 300 µM IAA with or without 5 µM of the AHR inhibitor CH-223191. Flow cytometry analysis of (A) IFNγ+ cells and (B) IL-17A+ cells among CD4+ T cells revealed no effect under IAA treatment or additional treatment with CH-223191 (n=8 per group; Two-way ANOVA with Šidák’s Multiple comparisons test; factor 1: IAA concentration; factor 2: CH-223191 addition). (C, D) Naïve CD4+ T cells from pwMS were cultured under co-stimulatory anti-CD3/anti-CD28 conditions in the presence or absence of 300 µM IAA with or without 5 µM of the AHR inhibitor CH-223191. (C) Flow cytometry analysis of IL-10+ cells among CD4+ T cells revealed a relative increase under IAA and no effect upon additional treatment with CH-223191 (n=9 per group; Two-way ANOVA with Šidák’s Multiple comparisons test; factor 1: IAA concentration; factor 2: CH-223191 addition). (D) The IAA induced increase in CYP1a1 gene expression was prevented by simultaneous addition of CH-223191 (n=4 per group; Two-way ANOVA with Šidák’s Multiple comparisons test; factor 1: IAA concentration; factor 2: CH-223191 addition).
